# Oridonin Delays Aging Through the AKT Signaling Pathway

**DOI:** 10.3389/fphar.2022.888247

**Published:** 2022-05-18

**Authors:** Yongpan An, Jie Zhu, Xin Wang, Xinpei Sun, Chunxiong Luo, Yukun Zhang, Yuwei Ye, Xiaowei Li, Abudumijiti Abulizi, Zhizhen Huang, Hang Zhang, Baoxue Yang, Zhengwei Xie

**Affiliations:** ^1^ Department of Pharmacology, School of Basic Medical Sciences, Peking University International Cancer Institute, Peking University, Beijing, China; ^2^ School of Physics, Peking University, Beijing, China; ^3^ Peking University-Yunnan Baiyao International Medical Research Center, Peking University Health Science Center, Peking University, Beijing, China; ^4^ Beijing Gigaceuticals Tech. Co. Ltd., Beijing, China

**Keywords:** oridonin, anti-aging, Akt, cell senescence, yeast, mice

## Abstract

Aging is a major risk factor for chronic diseases and disability in humans. Nowadays, no effective anti-aging treatment is available clinically. In this study, oridonin was selected based on the drug screening strategy similar to Connectivity MAP (CMAP) but upon transcriptomes of 102 traditional Chinese medicines treated cell lines. Oridonin is a diterpenoid isolated from Rabdosia rubescens. As reported, Oridonin exhibits a variety of pharmacological activities, including antitumor, antibacterial and anti-inflammatory activities. Here, we found that oridonin inhibited cellular senescence in human diploid fibroblasts (2BS and WI-38), manifested by decreased senescence-associated β-galactosidase (SA-β-gal) staining. Compared with the elderly control group, the positive cell rate in the oridonin intervention group was reduced to 48.5%. Notably, oridonin prolonged the lifespan of yeast by 48.9%, and extended the average life span of naturally aged mice by 21.6%. Our mice behavior experiments exhibited that oridonin significantly improved the health status of naturally aged mice. In addition, oridonin also delayed doxorubicin-induced cellular senescence and mouse senescence. Compared with the model group, the percentage of SA-β-gal positive cells in the oridonin treatment group was reduced to 59.8%. It extended the average lifespan of mice by 53.8% and improved healthspan. Mechanistically, we showed that oridonin delayed aging through the AKT signaling pathway and reversed the genetic changes caused by doxorubicin-induced cell senescence. Therefore, oridonin is a potential candidate for the development of anti-aging drugs.

## Introduction

Aging is characterized by the gradual loss of physiological integrity, leading to impaired functions, increasing susceptibility to diseases, and death. Aging is the leading risk factor for chronic diseases and disabilities in human society, which impacts social and health care expenditures. So far, research work on aging-related diseases is aimed at prolonging lifespan and extending the healthy period to delay, stop, or even reverse the aging process. Currently, there are just a few antiaging drugs on trial. These drugs include dasatinib + quercetin (Xu M et al., 2018), β-nicotinamide mononucleotide (NMN) (Zhang et al., 2016), metformin (Martin-Montalvo et al., 2013; Barzilai et al., 2016), rapamycin (Harrison et al., 2009; Li et al., 2014), etc. However, more drugs are required to slow aging and improve human life quality.

Currently, the screening of antiaging drugs is mainly based on known drug targets. However, this strategy is not efficient and has met bottlenecks. Given that aging is caused by multi-factors, we adopted a computational biology method CMAP (Connectivity Map) screening strategy. This screening method does not target specific targets, and screens drugs through a broad-spectrum comparison of gene expression profiles. It is well known that cell transcription changes after treatment with various compounds and gene expression profile reflect pathological characteristics, which is widely used to discover anticancer and antiobesity drugs (Liu et al., 2015). Some traditional Chinese medicines, such as berberine and EGCG (Dang et al., 2020; Huang et al., 2020), have been considered to delay aging. Therefore, we selected the genetic data of Chinese medicine compounds to screen the natural compounds with antiaging activity.

In this study, oridonin was selected based on CMAP. Experimental data showed that oridonin extended the replicative lifespan of yeast, delayed both replicative senescence and doxorubicin-induced cellular senescence, and prolonged the lifespan and natural senescence of doxorubicin-induced premature aging mice. Our experimental results suggest that oridonin may improve the healthy lifespan by regulating AKT signaling pathway. At the same time, it also affects the NLRP3 inflammation signaling pathway and reverses the genetic changes caused by doxorubicin-induced cell senescence.

## Materials and Methods

### Silybin, Oridonin, Sennoside A, Dioscin, Oxymatrine, Phillyrin, Schizandrin B and Doxorubicin

Silybin, oridonin, sennoside A, dioscin, oxymatrine, phillyrin, and schizandrin B were purchased from Topscience. Doxorubicin was purchased from HARVEYBIO. In *in vitro* experiments, DMSO was used to dissolve silybin, oridonin, sennoside A, dioscin, oxymatrine, phillyrin, and schizandrin B to prepare mother solutions, which were diluted for use. In *in-vivo* experiments, oridonin was dissolved in 0.5% sodium carboxymethyl cellulose.

### Yeast Mutant Strain Generation and Lifespan Detection

Both Sch9 yeast mutants and wild-type yeasts were derived from the library, Krogen lab. Standard SD medium (1% yeast extract, 1% bacterial protein, and 2% glucose) for all yeast strains cultured on a rotating shaker at 30°C and 250 rpm. Lifespan determination when yeast is in the logarithmic growth phase. As described in the previous reports (Xie et al., 2012), we observed the mother cells using a repeated microscope for 2 days. The survival curve is based on data collected from multiple experiments and corresponding controls. Survival analysis and cell cycle analysis were conducted using MATLAB.

### Cell Lines and Cell Culture

WI-38 and 2BS cells were provided by the National Institutes for Food and Drug Control. The cells were counted and inoculated for each passage at the same density, and incubated in MEM (Gibco) supplemented with 10% FBS (Gibco) in an incubator at 37°C and 5% CO_2_. WI-38 and 2BS cells before PD 30 were considered young. For the induction of premature senescence, WI-38 and 2BS cells were induced with 1 μM doxorubicin for 12 h. Oridonin treats cells for 24–48 h (Dang et al., 2020).

### Cell Viability Test

The cell viability was evaluated by the CCK-8 detection kit (Dojindo). 2BS or WI-38 cells were seeded into 96-well plates (5000/well), and then treated with drugs such as oridonin for 24 h. 1:10 diluted CCK-8 solution was added to the culture medium and incubated for 1 h at 37°C. Then a microplate reader (Biotek, MQX200) was used to measure the absorbance at 450 nm.

$$\text{Cell}\;\text{survival}\;\text{rate}\left(\text{\%}\right)=\left(\text{OD}\;{\text{treatment}-\text{OD}}_{\text{blank}}\right)\text{/}\left({\text{OD}}_{\text{control}}{-\text{OD}}_{\text{blank}}\right)\text{×}100\text{\%}$$

### SA-β-Gal Staining

The cells were washed twice with PBS, then fixed with 4% formaldehyde at room temperature for 15 min, and washed three times. Then the cells were stained with a staining buffer {[1 mg/ml 5-bromo-4-chloro-3-indolyl-β-d-galactopyranoside (X-gal) at 37°C, 40 mM citric acid/Sodium phosphate, pH 6.0] stain overnight, 5 mM potassium ferrocyanide, 5 mM potassium ferricyanide, 150 mM NaCl, 2 mM MgCl_2_}, and then analyzed according to the instructions provided (CST, 9860S). Use ImageJ software to count SA-β-gal positive cells.

### Western Blot

WI-38 cells were first induced with 1 μM doxorubicin for 12 h, and then treated with oridonin or DMSO-containing medium for 48 h. WI-38 cells homogenized in RIPA lysis buffer (Thermal Scientific) with protease inhibitors (Roche) and 1% phosphatase inhibitors (Applygen). The protein extracts were homogenized using a Dounce homogenizer and spun at 12,000 g for 20 min at 4°C. BCA analysis (Pierce) was used for protein quantification, and then SDS-PAGE was used to separate the proteins and then transfer to a PVDF membrane (Amersham Biosciences). The botting membranes were incubated with primary antibodies against ACTIN, p-FOXO1, FOXO1, p21, p53, IL-1α or IL-1β (ProteinTech), IL-6 (ABclonal), and IL-8 (Immunoway) overnight at 4°C. Secondary goat anti-rabbit or anti-mouse IgG (Scicrest) was added for blotting. ECL Plus kit (Amersham Biosciences) was used for visualization. The optical density of each band was used to quantify relative protein level.

### RNA Extraction and qPCR

TRIzol was used to lyse cells, and then isopropanol and chloroform were used to extract RNA. Using cDNA synthesis kit, a total of 1 μg RNA was used for cDNA reverse transcription (Abmgood). Real-time qPCR was used to evaluate the target gene’s expression and the expression level was normalized to GAPDH. Supplementary Table S1 lists all primers.

### Obtaining and Lifespan Testing of Naturally Aged Mice

All naturally aged mice were purchased from SPF Biotechnology Co., Ltd., Beijing, China. The mice were kept in an environment with minimum pressure and standard conditions, namely constant temperature and a light/dark cycle of 12:12 h. The animals were randomly assigned to the control group and the treatment group.

Life expectancy test: 16 months old, male C57BL/6J mice were intraperitoneally injected with oridonin for 1 week every month; the dosage was 2 mg/kg for 6 months, and the control group was given the same volume of 0.5% sodium carboxymethyl cellulose. The death time of mice was recorded.

### Behavioral Testing

Grip strength test: the mice were placed on top of the grip strength meter, so they grasped the grid with all four paws. The meter was set to “peak tension” (T-PK) mode, the grip of 5 trials was recorded, and the final data were the maximum grip (g).

Learning and memory test: on the first day, the mouse was placed in a frame 40 cm long, 40 cm wide, and 40 cm high to adapt to the environment for 5 min. On the second day, two identical objects are placed in the frame. The mouse explores autonomously for 5 min on the third day. One of the objects was replaced and the time was recorded when the mouse autonomously analyzed new and old objects. Mouse score calculated as DI = (NEWtime-OLDtime)/(NEWtime + OLDtime).

Working memory ability: Y maze test was used. The mouse was placed at the end of one arm. The order was recorded in which the mouse entered each component within 5 min. Alternation is defined as entering three components continuously, such as (1, 2, 3 or 1, 3, 2), the maximum alternation is the sum of the number of component advances -2, and then the percentage is calculated = alternation/maximum alteration×100%.

### Doxorubicin Induces Acquisition and Life Span Detection in Aging Mice

All mice were purchased from SPF Biotechnology Co., Ltd., Beijing, China. The mice were kept in an environment with minimum pressure and standard conditions, namely constant temperature and a light/dark cycle of 12:12 h. The animals were randomly assigned to the control group and the treatment groups. 2-month-old male C57BL/6J mice were injected intraperitoneally with doxorubicin (10 mg/kg) every 5 days for 3 times. Oridonin (2 mg/kg) was injected intraperitoneally every 3 days. The control group was given 0.5% sodium carboxymethyl cellulose, and the death of the mice was recorded.

### Tissue Morphological Staining

Masson’s trichrome staining: to test heart tissue fibrosis, Masson’s trichrome staining kit (Solarbio, G1340) was used. The sections fixed with formalin and embedded in paraffin were dealkylated and rehydrated with 100% alcohol, 95% alcohol and tap water. Then the sections were stained according to the manufacturer’s instructions. Images were collected using a strong light microscope (×10 magnification objective lens) (OLYMPUS, BX43), and analyzed by ImageJ software (NIH) to measure the area of fibrosis and the total area. The percentage of fibrosis was calculated by dividing the fibrosis-positive area by the entire area. Finally, the statistical results are normalized.

H&E staining: The kidney was fixed with 4% formaldehyde to test the tissue morphology, followed by paraffin embedding and sectioning. The sections were stained with H&E for morphological analysis. The renal tubule damage was scored. When the brush border of the renal tubule was lost, the lumen was dilated, and the epithelial cells were flattened and atrophied, the damaged tubules were counted. Scores were calculated according to the following criteria: 0 = normal; 1=< 20%; 2 = 20–40%; 3 = 40–60%; 4 = 60–80%; and 5≥ 80%.

### RNA-Seq

WI-38 cells were first induced with 1 μM doxorubicin for 12 h, and then treated with oridonin or DMSO-containing medium for 48 h. Total RNA was extracted from the tissue using TRIzol® Reagent according to the manufacturer’s instructions (Invitrogen) and genomic DNA was removed using DNase I (TaKara). Then RNA quality was determined by 2100 Bioanalyser (Agilent) and quantified using the ND-2000 (NanoDrop Technologies). Only a high-quality RNA sample (OD260/280 = 1.8–2.2, OD260/230 ≥ 2.0, RIN≥ 6.5, 28S:18S ≥ 1.0, >2 μg) was used to construct the sequencing library. After quantified by TBS380, paired-end RNA-seq sequencing library was sequenced with the Illumina HiSeq X ten/NovaSeq 6000 sequencer (2 × 150 bp read length). RNA sequencing and raw data quality control were performed by Shanghai Majorbio Bio-pharm Technology Co., Ltd. Two threshold criteria for gene set selection: fold up or down-regulation fold change> 1.20 and an associated *p*-value of less than 0.05.

### Statistical Analysis

Wilcoxon rank-sum test was used to compare life span differences. Evaluate the results through a two-tailed Student’s *t*-test or analysis of variance to compare two and more samples, respectively. The data is the mean ± SEM. *p* < 0.05 is the significance threshold. *p < 0.05, **p < 0.01, ***p < 0.001, ****p < 0.0001. n.s., not significant. Unless otherwise stated, the results are based on a minimum of three independent experiments. Unless otherwise stated, use GraphPad Prism for statistics.

## Results

### Oridonin Exhibits Inhibitory Activity on Cellular Senescence

**FIGURE 1:**
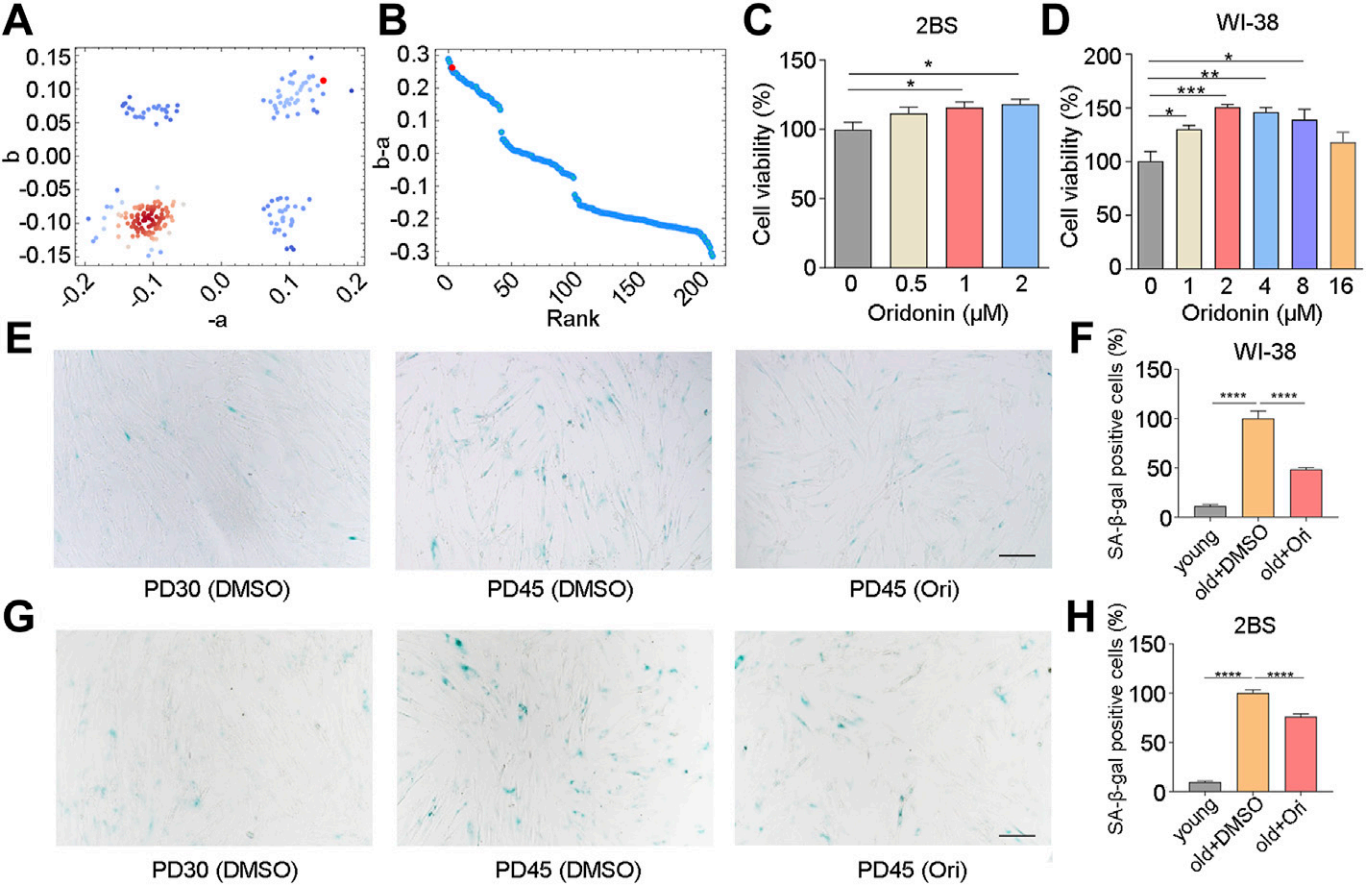
Oridonin screened by CMAP delays cell senescence. **(A** **–B)** Prediction of anti-aging efficacy using CMAP. Red dots indicate tested small molecules. **(C)** The effect of different concentrations of oridonin on the proliferation of PD45 2BS cells was measured by the CCK-8 assay. **(D)** The effect of different concentrations of oridonin on the proliferation of PD45 WI-38 cells was measured by the CCK-8 assay. **(E)** SA-β-gal staining of PD45 WI-38 cells treated with 2 µM oridonin or DMSO for 48 h, Bar = 200 µm. **(F)** Quantification of the rate of SA-β-gal positive cells in PD45 WI-38 cells. **(G)** SA-β-gal staining of PD45 2BS cells treated with 2 µM oridonin or DMSO for 48 h, Bar = 200 µm. **(H)** Quantify the proportion of SA-β-gal positive cells in PD45 2BS cells (C-H, *n* ≥ 3 for each group). Data represent the mean ± SEM. one-way ANOVA or Student’s t-test determined *p* values. (*p < 0.05, **p < 0.01, ***p < 0.001, ****p < 0.0001).

Considering that muscle is the most abundant tissue in the human body, we analyzed the transcriptional changes of muscle tissue of the elderly compared with the muscle tissue of the young, which was carried out by Todd R Golub et al. (Lamb et al., 2006). Gene set enrichment analysis calculated the anti-aging score (Figures 1A,B). The results revealed 7 top-ranked and commercially available compounds from the subset, including silybin, oridonin, sennoside A, dioscin, oxymatrine, phillyrin, and schizandrin B of natural compounds. To check the antiaging effects of these compounds, we firstly examined the cell viability in human embryonic lung diploid cells 2BS treated with different concentrations of these compounds, and found that oxymatrine, schizandrin B and oridonin significantly enhanced the viability of senescent cells (Figure 1C and Supplementary Figures S1A,B), while silybin, phillyrin, sennoside A and dioscin did not improve the viability (Supplementary Figures S1C–F). To determine whether this observation was limited to a specific cell type, the effect of schizandrin B and oridonin on cell viability was further verified in another senescent cell line embryonic lung fibroblast WI-38. The results demonstrated that oridonin significantly improved the cell viability of senescent WI-38 cells in a dose-dependent manner (Figure 1D), while schizandrin B did not improve the cell viability (Supplementary Figure S1G). Based on these assessments, oridonin, the top-ranked compound from the CMAP analysis (red dots as shown in Figures 1A,B), was chosen as a potent natural anti-aging compound for further study.

To validate the effect of oridonin, we examined the senescence-associated β-galactosidase (SA-β-gal) activity in WI-38 and 2BS cells, which is a widely used marker in different types of senescent cells for characterizing aging *in vivo* and *in vitro*. Young WI-38 cells (PD30) had a flat, scattered appearance and were evenly spaced apart, while old cells (PD45) showed senescence characteristics (Figure 1E). Compared with PD45 cells in the elderly control group, only 11.3% of SA-β-gal positive cells were observed in PD30 cells, and the percentage of SA-β-gal positive cells in oridonin-treated WI-38 cells (PD45) was 48.5% (51.5% reduction, Figures 1E,F). Then we used 2BS cells to verify further that oridonin inhibited cellular senescence. Similarly, young 2BS cells (PD30) had a flat, spread appearance and even spacing, while cells in senescent cells (PD45) showed the characteristics of senescence, granular cytoplasm and accumulation of inclusions (Figure 1G). Compared with the PD45 cells, only 9.7% of SA-β-gal positive cells were observed in PD30 cells, and the proportion of SA-β-gal positive cells in senescent cells treated with oridonin was reduced to 76.1% (Figures 1G,H). Collectively, these results demonstrate that oridonin identified by CMAP analysis effectively maintains the viability of senescent cells and delays the replicative senescence progression.

### Oridonin Prolongs Yeast Replicative Lifespan

Developing anti-aging drugs is a tedious and time-consuming process, and only long-term life tests can obtain meaningful results. The yeast strain *Saccharomyces cerevisiae* (Xie et al., 2015) is one of the commonly used model organisms for aging research. The aging mechanism in yeast and other model organisms is also highly conserved. Budding yeast shows progressive senescence similar to that of mammals (Gershon and Gershon, 2000). In our attempt to determine the replicative lifespan of yeast, an automated device based on a microfluidic chip described previously was employed (Jo et al., 2015), which can measure the lifespan of yeast within 3 days.

**FIGURE 2:**
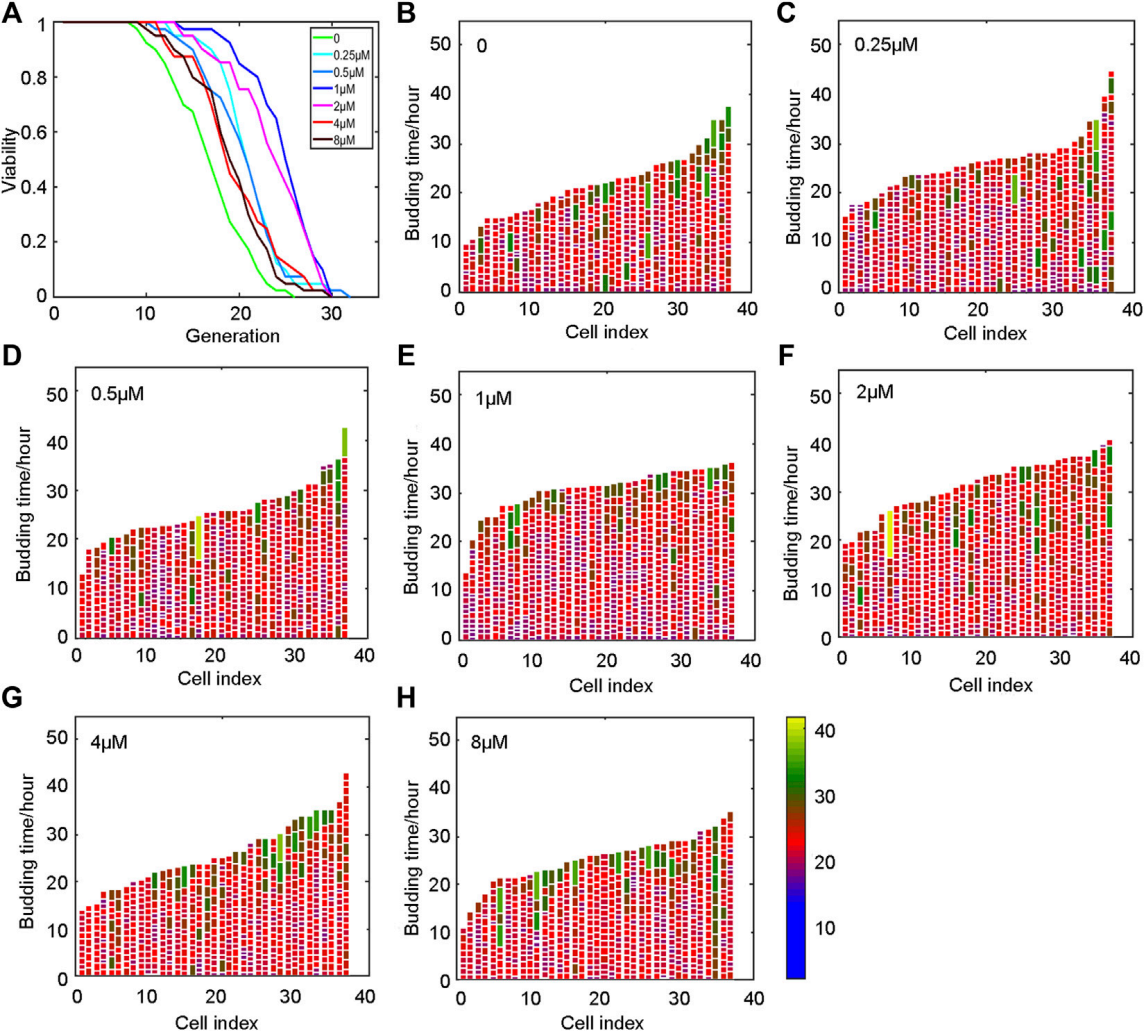
Oridonin prolongs the lifespan of yeast. **(A)** Oridonin extends the replication life of yeast. **(B–** **H)** Germination diagrams of wild-type and oridonin-treated mother cells show cell cycle duration and heterogeneity (**A–H**, *n* = 40 for each group) (see index color scale. Duration is 1.4 h or less. Cell cycle is colored in color purple). The x-axis shows a single parent cell as a vertical bar, while budding events are shown as white horizontal partitions.

Yeast was incubated in SD medium for 20 h, and then collected and loaded on the chip. The lifespan was monitored when the oridonin concentration was 0, 0.25, 0.5, 1, 2, 4, and 8 μM. The experimental results showed that oridonin led to a robust lifespan extension of 14.6–48.9% in the BY4741 strain in a dose-dependent manner (Figure 2A) compared with control media. In addition, oridonin treatment significantly reduced the number of cells with longer cell cycle duration (Figures 2B–H), thus decreasing the heterogeneity of cell cycle length, and prolonged lifespan.

### Oridonin Prolongs Lifespan and Healthspan in Mice

**FIGURE 3:**
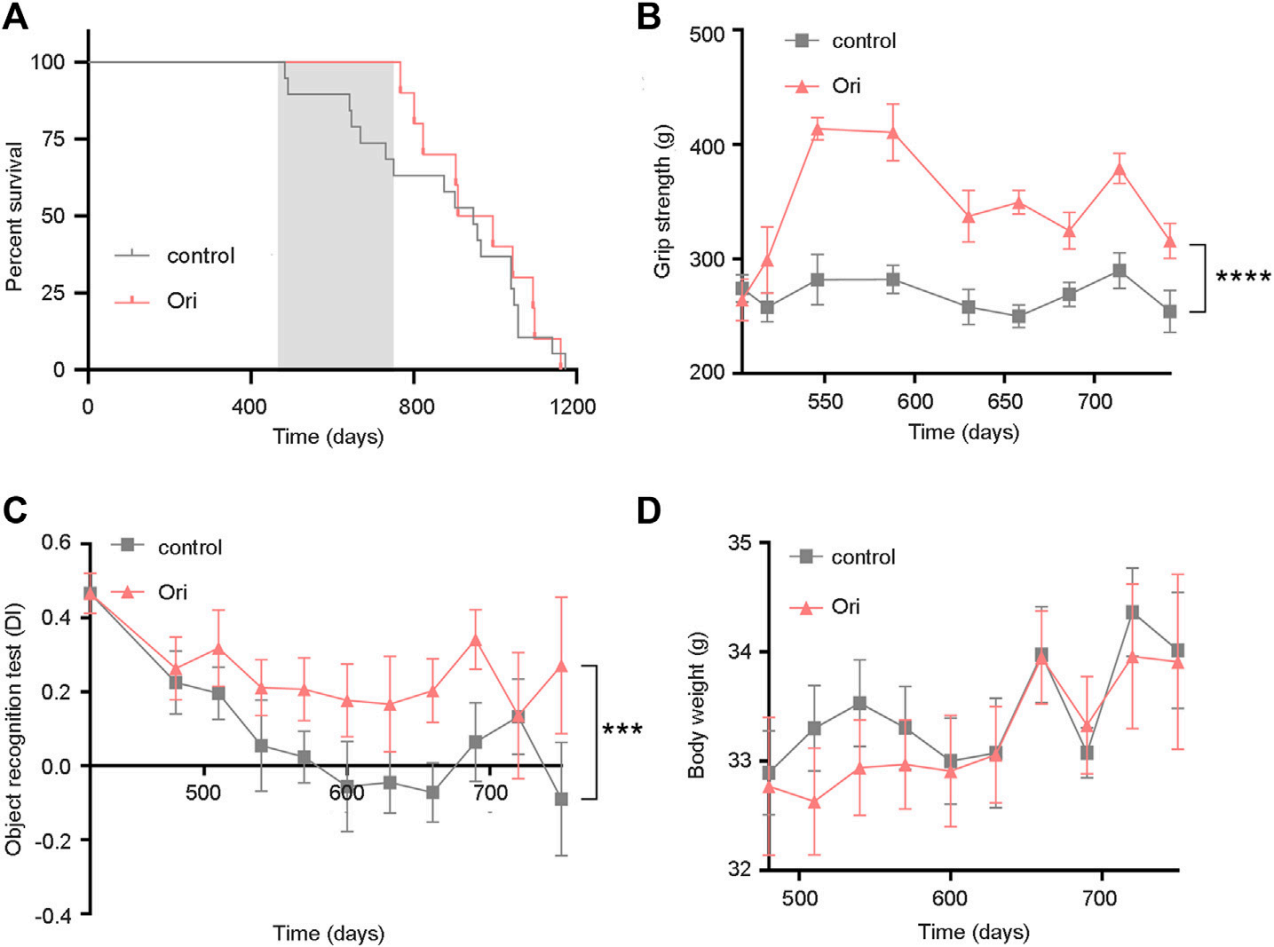
Oridonin prolongs the lifespan of mice and increases healthy lifespan. 16-month-old C57BL/6J mice were treated with vehicle or oridonin once a month for 6 months. **(A)** The survival curve of male mice treated with oridonin (Ori) (2 mg/kg) or vehicle control (the shadow is the period of administration. Control *n* = 19, oridonin 2 mg/kg *n* = 10). **(B)** Oridonin (Ori) (2 mg/kg) improves the grip of male mice (Control *n* = 19, oridonin 2 mg/kg *n* = 10). **(C)** Oridonin (Ori) improves the learning and memory ability of male mice (new object recognition experiment, DI: Discrimination index) (control *n* = 19, oridonin 2 mg/kg *n* = 10). **(D)** Detect the weight change of male mice under the intervention of oridonin (Ori) (2 mg/kg) (control *n* = 19, oridonin 2 mg/kg *n* = 10). Data represent the mean ± SEM. *p* values were determined by one-way ANOVA, two-way ANOVA or Student’s *t-test*. (*p < 0.05, **p < 0.01, ***p < 0.001, ****p < 0.0001).

To further test whether oridonin can prolong the lifespan of mice, we selected 16-month-old C57BL/6J male mice to conduct lifespan and healthy lifespan tests under the intervention of oridonin. During the experiment, the oridonin treatment group extended the average life span of mice from 394 to 479 days (85 days, approximately 21.6% extension). Throughout the life process, the oridonin treatment group extended the average life span of mice from 874 to 959 days (85 days, approximately 9.7% extension) (Figure 3A). Moreover, we evaluated the physical function of old mice treated with oridonin through functional tests. Compared with the control group, oridonin treatment significantly improved grip strength (Figure 3B) and learning memory ability (Figure 3C). There was no significant difference in the average weekly weight between the control and treatment groups (Figure 3D), indicating the safety of taking 2 mg/kg of oridonin regularly. These results suggest that oridonin improves the lifespan and health of naturally aging mice.

### Oridonin Suppresses Doxorubicin-Induced Premature Senescence

**FIGURE 4:**
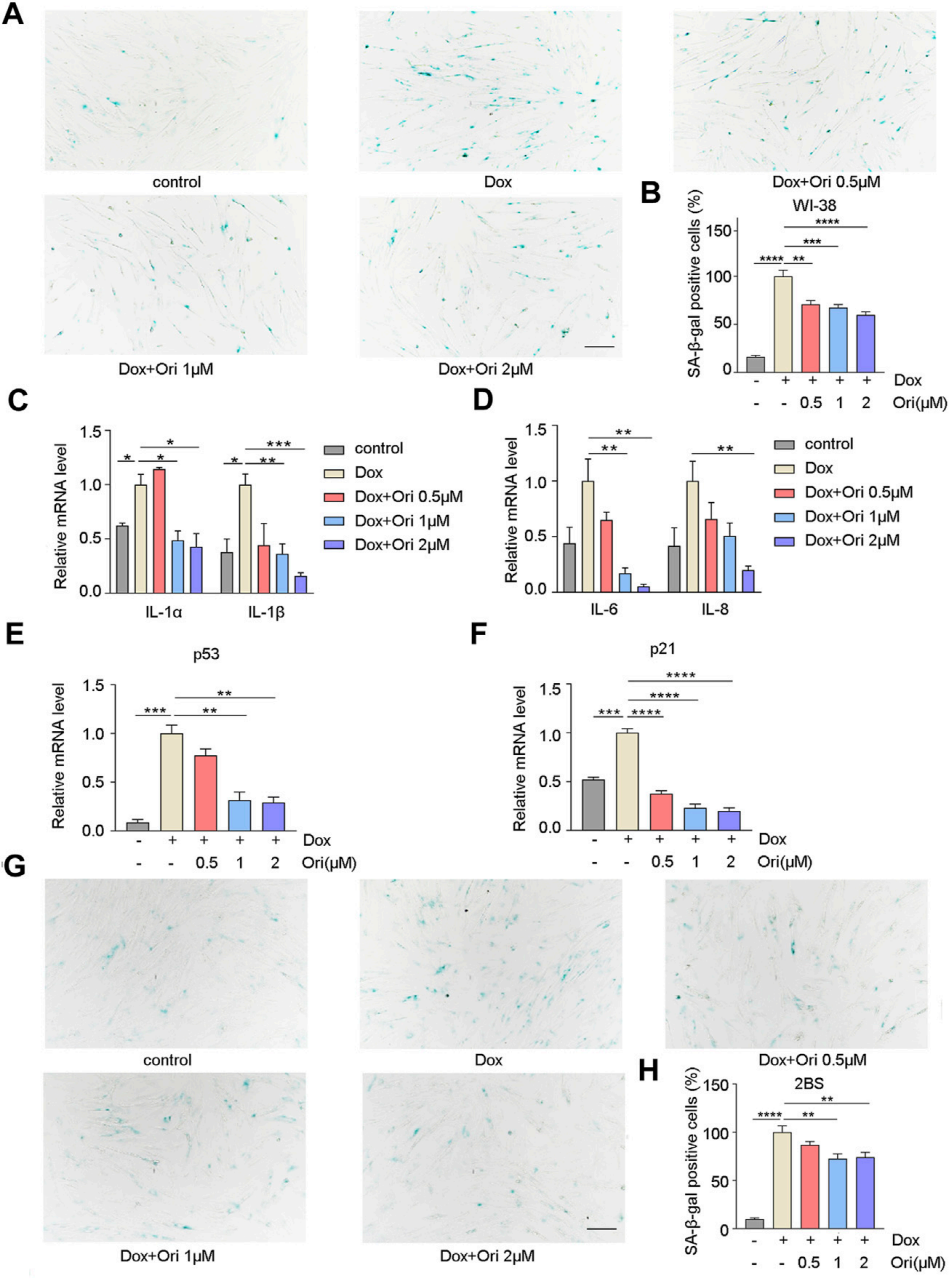
Oridonin delays cell senescence induced by doxorubicin. 1 μM doxorubicin-induced PD30 2BS or WI-38 cells for 12 h. **(A)** SA-β-gal staining of doxorubicin-induced WI-38 cells treated with 0.5, 1, 2 µM oridonin or DMSO carrier for 48 h, Bar = 200 µm. **(B)** Quantify the proportion of SA-β-gal positive cells in WI-38 cells treated with different treatments. **(C** **–D)** Treatment of doxorubicin-induced WI-38 cells with 0.5, 1, 2 µM oridonin or DMSO carriers for 36 h of IL-1α, IL-1β, IL-6, and IL-8 mRNA levels. **(E** **–F)** The changes of p53 and p21 mRNA levels in WI-38 cells induced by doxorubicin were treated with 0.5, 1, 2 µM oridonin or DMSO for 36 h. **(G)** SA-β-gal staining of doxorubicin-induced 2BS cells treated with 0.5, 1, 2 µM oridonin or DMSO carriers for 48 h, Bar = 200 µm. **(H)** Quantification of SA-β-gal positive cell rate in 2BS cells of different treatments (**A−** **H**, *n* ≥ 3 for each group). Data represent the mean ± SEM. one-way ANOVA or Student’s t-test determined *p* values. (*p < 0.05, **p < 0.01, ***p < 0.001, ****p < 0.0001).

Off-target toxicity limits the maximum tolerated dose of chemotherapeutic drugs and causes long-term health problems in cancer survivors, including accelerated aging (Henderson et al., 2014). The expression of the senescence markers p53 and p21 is upregulated, which leads to the generation of senescence-associated secretory phenotype (SASP) (Van Deursen, 2014; Childs et al., 2017; Wakita et al., 2020). Since chemotherapy can induce senescence (Ewald et al., 2010), we further explored whether oridonin could delay cellular senescence caused by chemotherapy drug doxorubicin. PD30 WI-38 and 2BS cells were treated with 1 μM doxorubicin for 12 h to detect whether oridonin could suppress the cellular senescence. In WI-38 cells, the percentage of SA-β-gal positive cells in the control group was 16.2% of the model group, and the rate of SA-β-gal positive cells in the oridonin-treated model group decreased in a dose-dependent manner (Figures 4A,B). At the same time, we also tested senescence markers, including p21, p53, and SASP. RT-qPCR results showed that compared with the control group, the mRNA and protein expression of IL-1α, IL-1β, IL-6, IL-8, p21, and p53, were upregulated in the model group. After oridonin treatment, the expression of these genes was downregulated (Figures 4C–F and Supplementary Figures S2A–E). Similarly, in 2BS cells, compared with the model group, the SA-β-gal positive cell rate in the control group was 10.0%, and the SA-β-gal positive cell rate in the oridonin-treated model group was significantly reduced (Figures 4G,H). The results indicate that oridonin delays cell senescence induced by doxorubicin.

### Oridonin Prolongs the Lifespan and Health Span in Premature Aging Mice

**FIGURE 5:**
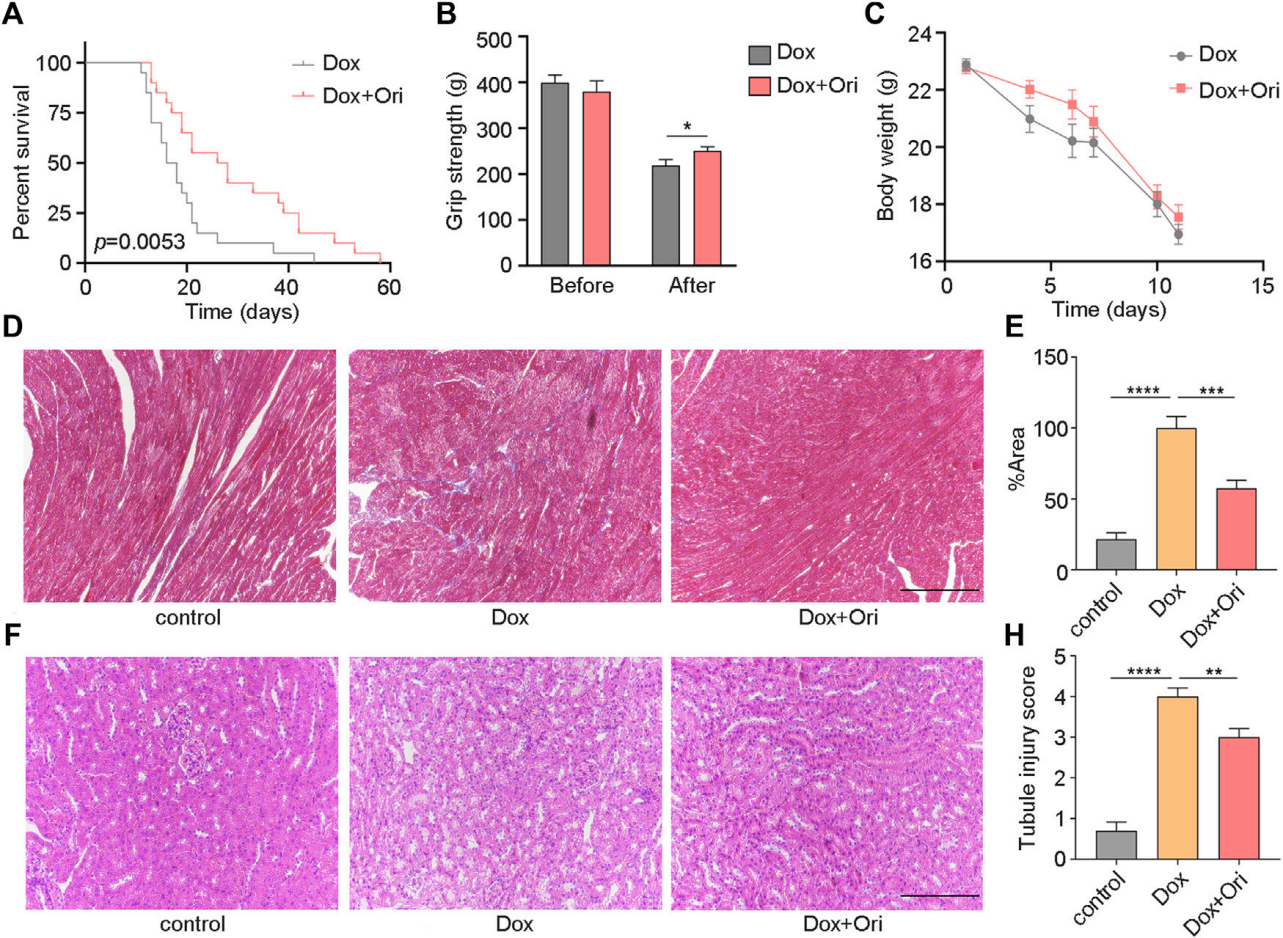
Oridonin prolongs the lifespan and healthy lifespan of premature aging mice. **(A)** Oridonin (Ori) (2 mg/kg) prolongs the lifespan of mice with premature aging induced by doxorubicin (model *n* = 19, oridonin 2 mg/kg *n* = 10, P= 0.0053). **(B)** The mouse grip test (left) before the doxorubicin model and oridonin (Ori) intervention, and the mouse grip test (right) 2 weeks after the doxorubicin model and oridonin (Ori) intervention (*n* = 12–20). **(C)** Doxorubicin-induced senescence in mice, and body weight was detected under the intervention of oridonin (Ori) (*n* = 12–20). **(D–** **E)** Masson’s trichrome staining was used to detect the cardiac morphology, fibrosis and quantification of mice in the control group, doxorubicin-induced aging and oridonin (Ori) intervention groups (*n* = 6 per group), Bar = 100 µm. **(F)** HE staining to detect the kidney morphology of mice in the normal control group, doxorubicin-induced aging and oridonin (Ori) intervention groups (*n* = 6 per group), Bar = 100 µm. **(H)** Kidney injury score (*n* = 6 per group). Data represent the mean ± SEM. *p* values were determined by one-way ANOVA, Student’s *t-test* or. Log-rank (Mantel-Cox) test. (*p < 0.05, **p < 0.01, ***p < 0.001, ****p < 0.0001).

We speculated that oridonin could extend the lifespan of an accelerated aging mouse model induced by doxorubicin (Dang et al., 2020; Wiley et al., 2021). In this experiment, 8-week-old male C57BL/6J mice have induced senescence with a dose of 10 mg/kg doxorubicin. We found that oridonin treatment extended the average life span of doxorubicin-induced aging mice from 19 to 29 days (10 days, approximately 53.8% extension) compared to the control group (Figure 5A). Notably, mice treated with oridonin had significantly increased grip strength than the control group (Figure 5B), but there was no significant change in body weight (Figure 5C). Doxorubicin has been reported to induce nephrotoxicity and cardiotoxicity (Majumder et al., 2018; Galan-Arriola et al., 2019; Guerrero et al., 2019; Dang et al., 2020; Yang et al., 2020). Masson’s trichrome staining results showed that the degree of cardiac fibrosis of the doxorubicin-treated group was significantly increased compared to control mice, and oridonin treatment significantly attenuated doxorubicin-induced myocardial fibrosis (Figures 5D,E). Hematoxylin and eosin (H&E) staining conducted a histomorphological analysis of the kidney. Compared with the control group, the model group had noticeable damage to the renal tubules, while the oridonin treatment group had significantly less damage (Figures 5F–H). These results suggest that oridonin prolongs the lifespan, improves health span, and improves tissue morphological changes in doxorubicin-induced aging mice.

### Oridonin Extends Lifespan Through AKT and NLRP3

**FIGURE 6:**
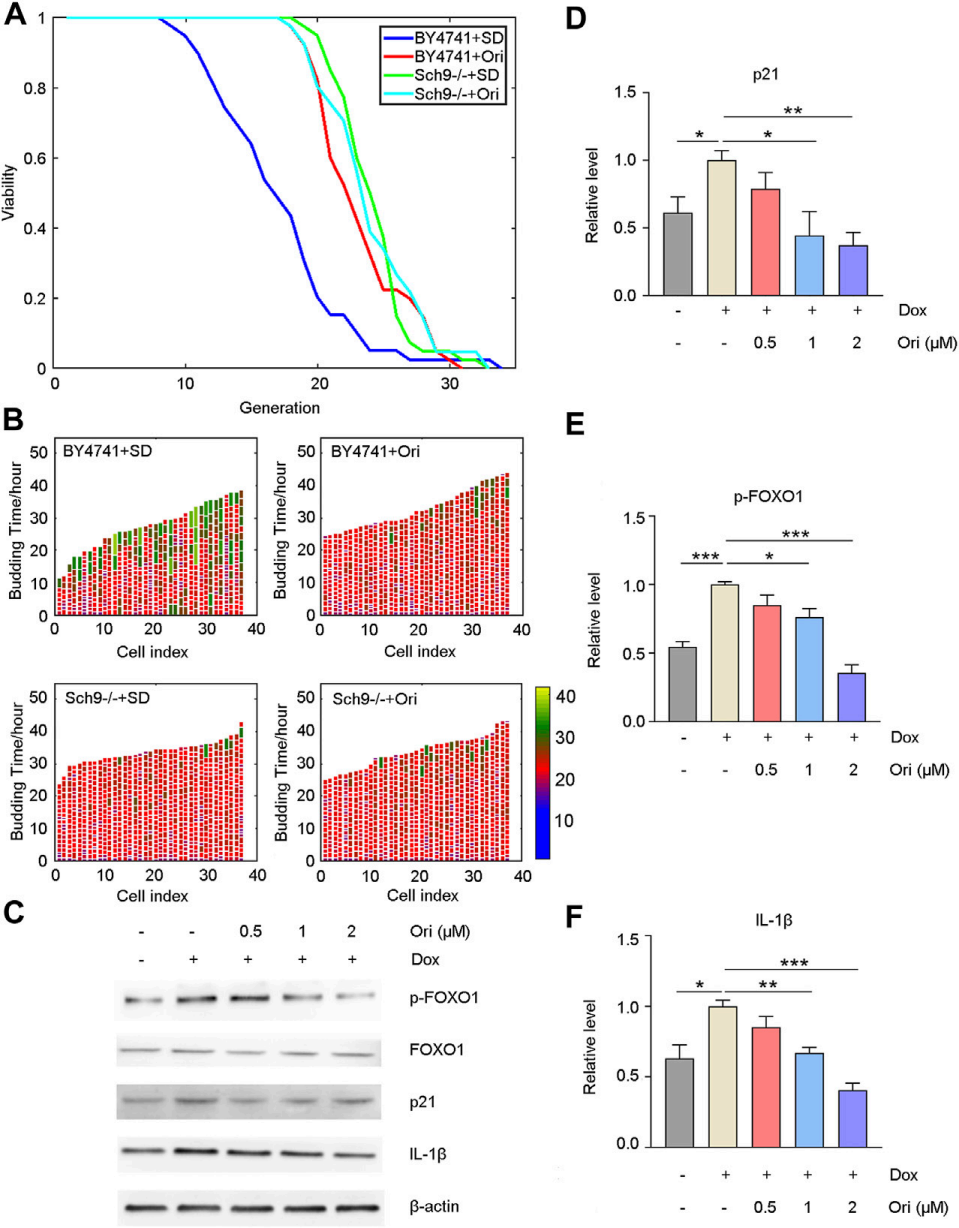
Oridonin delays aging through AKT and NLRP3. **(A)** Wild-type cells treated with oridonin (1 μM) (BY4741 + Ori) have a longer replication life span than wild-type cells in SD medium (BY4741 + SD). Compared with the Sch9 knockout strain (Sch9−/− + SD), the Sch9 knockout strain treated with oridonin (1 μM) (Sch9−/− + Ori) did not extend its lifespan. At the same time, compared with the wild type, the Sch9 knockout strain has a longer life span. **(B)** Wild-type (BY4741 + SD), oridonin-treated wild-type cells (BY4741 + Ori), Sch9 knockout strain (Sch9−/− + SD), and oridonin-treated Sch9 knockout strain (Sch9−/− + Ori), showing cell cycle duration and heterogeneity (see index color scale; cell cycles with a duration of 1.4 h or less are colored in purple). The x-axis shows a single mother cell as a vertical bar, while budding events are shown as horizontal white partitions (**A–B**, *n* = 40 for each group). **(C)** Western blot analysis of p-FOXO1, FOXO1, p21, and IL-1β protein expression in the control group, the doxorubicin-induced aging model group, and the 0.5, 1, 2 µM oridonin treatment group. **(D–** **F)** Quantification of P-FOXO1, FOXO1, p21, and IL-1β protein expression in the control group, doxorubicin-induced aging model group, 0.5, 1, and 2 µM oridonin treatment group analyzed by Western blot (**C–F**, *n* ≥ 3 for each group). Data represent the mean ± SEM. one-way ANOVA or Student’s t-test determined *p* values. (*p < 0.05, **p < 0.01, ***p < 0.001, ****p < 0.0001).

It has been reported that oridonin is an inhibitor of AKT (Song et al., 2018). We first knocked out the homologous gene Sch9 of AKT in yeast to explore how oridonin plays an antiaging effect and possible targets. Oridonin treatment failed to extend its lifespan in the Sch9 knockout strain. At the same time, the Sch9 knockout strain had a more prolongated lifespan than the wild type. The strain’s lifespan is extended by 42.3% (Figures 6A,B). This result indicates that oridonin prolongs the lifespan of yeast through Sch9, and knocking out Sch9 can prolong the lifespan of yeast.

Western blot detected downstream signaling pathways of AKT and aging-related proteins to confirm that oridonin functions through AKT in human cells. Compared with the control group, the doxorubicin-induced senescent cell model group had upregulated FOXO1 phosphorylation level and p21 expression. In contrast, FOXO1 phosphorylation and p21 expression were downregulated in the oridonin-treated cells, thereby prolonging lifespan (Figures 6C–E). Oridonin has been reported to act directly on NLRP3 inflammasomes and block the assembly of NLRP3 inflammasomes (He et al., 2018). Therefore, we used the Western blot to detect its downstream signal pathways, such as IL-1β. As expected, compared with the control group, the expression of IL-1β in the doxorubicin-induced senescent cell model group was upregulated. In contrast, the expression of IL-1β in the oridonin-treated group was downregulated (Figures 6C–E). These results indicate that oridonin exerts anti-aging effects through AKT and NLRP3.

### Oridonin Reverses the Gene Changes Caused by Doxorubicin-Induced Cell Senescence

**FIGURE 7:**
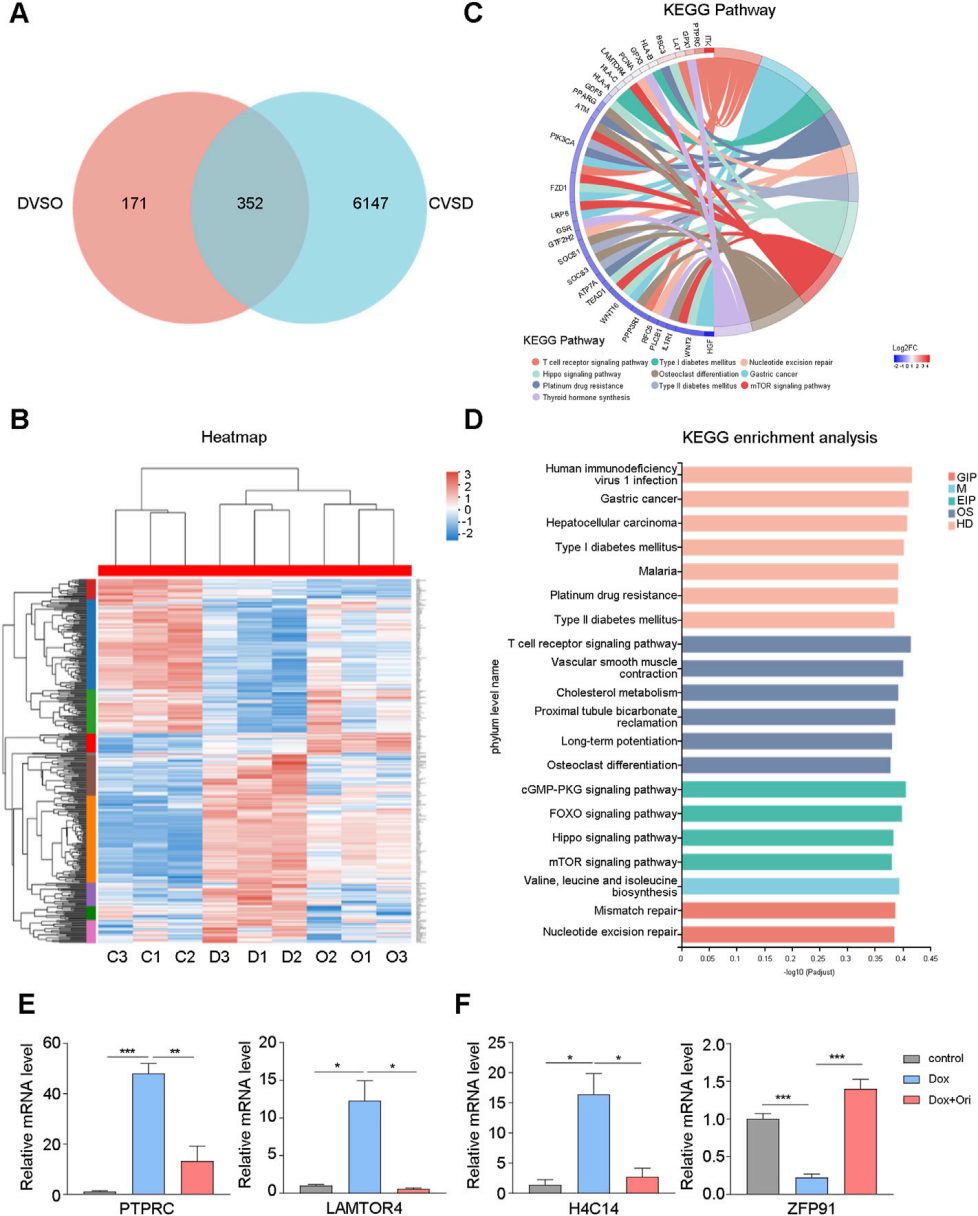
Oridonin reverses the gene changes caused by doxorubicin-induced cell senescence. WI-38 cells were first induced with 1 μM doxorubicin for 12 h, and then treated with oridonin or DMSO-containing medium for 48 h. **(A)** The differential gene (CVSD) produced by doxorubicin-induced cell senescence and the differential gene (DVSO) produced by oridonin treatment of doxorubicin-induced senescent cells were used for Venn analysis. **(B)** Gene heatmap: the intersection gene of the differential gene (CVSD) produced by doxorubicin-induced cell senescence and the differential gene (DVSO) produced by oridonin treatment doxorubicin-induced senescent cells. **(C)** Chord plot of KEGG terms: the intersection of the differential gene (CVSD) produced by doxorubicin-induced cell senescence and the differential gene (DVSO) produced by oridonin treatment of doxorubicin-induced senescent cells. **(D)** KEGG enrichment analysis: the intersection of the differential gene (CVSD) produced by doxorubicin-induced cell senescence and the differential gene (DVSO) produced by oridonin treatment doxorubicin-induced senescent cells. Different colors indicate the 7 branches of the KEGG metabolic pathway, namely metabolism (M), genetic information processing (GIP), environmental information processing (EIP), cellular processes (CP), biological systems (OS), and human diseases (HD), Drug Development (DD). **(E)** Quantitative PCR analysis of gene expression. **(F)** Quantitative PCR analysis of the histones and ubiquitin ligases (**A–F**, *n* = 3 for each group). Data represent the mean ± SEM. one-way ANOVA or Student’s t-test determined *p* values. (*p < 0.05, **p < 0.01, ***p < 0.001, ****p < 0.0001).

To further study whether oridonin also affects other signaling pathways. In WI-38 cells, we used RNA-seq to detect genetic changes in young cells, doxorubicin-induced senescent cells, and oridonin-treated doxorubicin-induced senescent cells. Doxorubicin-induced cellular senescence (CVSD) and oridonin intervention (DVSO) had 352 genes that changed in common (Figure 7A). The heatmap found that doxorubicin caused upregulation or downregulation of genes, and oridonin intervention reversed most genes’ changes (Figure 7B). Next, we performed GO enrichment analysis based on these 352 genes, and found the main modifications in cell signal transduction (Supplementary Figure S3A). Therefore, we performed a KEGG enrichment analysis, and found that the FOXO signaling pathway, mTOR signaling pathway had changed (Figures 7C,D), noting that these two signal pathways are also directly related to AKT. Additionally, we also found changes in the T cell receptor, cGMP-PKG, and Hippo signaling pathways (Figures 7C,D). Finally, we used the differentially expressed genes caused by doxorubicin-induced cell senescence (CVSD) and the differentially expressed genes caused by oridonin treatment of senescent cells (DVSO) to perform GO and KEGG enrichment analysis. The same signal transduction-related genes had major changes (Supplementary Figures S3B–D). Oridonin treatment also changed the AGE-RAGE, NF-κB, and TNF signaling pathways (Supplementary Figure S3D). The RT–qPCR results also supported these findings (Figure 7E). Surprisingly, among the differentially expressed genes caused by oridonin, we found that the top-ranked genes included histone and ubiquitin ligase-related genes, and RT–qPCR also confirmed this finding (Figure 7F). This result indicates that oridonin may delay aging by influencing epigenetics. These results indicate that oridonin reversed the genetic changes caused by doxorubicin-induced cell senescence.

## Discussion

In this study, oridonin was found, selected based on a TCM-CMAP, to prolong the lifespan of yeast, and inhibit replicative senescence and doxorubicin-induced senescence in human cells, and extend the lifespan of naturally aged mice and doxorubicin-induced aging mice and improve healthspan. Oridonin did not extend the lifespan of the Sch9 knockout strain, and the Sch9 knockout strain had a longer lifespan compared to the wild-type strain. These results indicate that oridonin exerts antiaging effects by inhibiting AKT signaling.

Numerous antiaging compounds have been identified, including NAD^+^ supplements: nicotinamide riboside (NR) and nicotinamide mononucleotide (NMN), but further research is still needed to determine whether NR or NMN have Beneficial effects; senolytics: Satinib and quercetin, but there are still some unanswered questions about this strategy of targeting senescent cells. Most of the senolytics discovered so far may affect non-senescent cells, and senolytics can lead to stem cell exhaustion (Kirkland and Tchkonia, 2017; Partridge et al., 2020); mTOR inhibitor: rapamycin, but rapamycin induces hyperglycemia, hyperglycemia Hyperlipidemia, renal toxicity, poor wound healing, decreased platelet count, and immunosuppression (Augustine et al., 2007; De Oliveira et al., 2011); AMPK activators: metformin and aspirin, etc. The side effects of metformin are also apparent, and some patients may experience diarrhea, abdominal distension, etc. (Soukas et al., 2019), aspirin also increases the risk of gastrointestinal bleeding (Mcneil et al., 2018). Therefore, more anti-aging drugs still need to be discovered, and the TCM compound library still has great potential for mining. At the same time, we are the first to apply the CMAP screening strategy to the screening of anti-aging drugs, and provide suggestions for anti-aging drug screening.

Oridonin is the main component of Rubescens that is commonly used in Chinese medicine to treat inflammatory diseases (Chen et al., 2009). Oridonin has attracted widespread attention due to its various pharmacological properties, including antitumor (Zhou et al., 2007; Bao et al., 2014), and inflammatory field (Ku and Lin, 2013), and antibacterial properties (Xu et al., 2014). Here, we used yeast, cell and mouse models to prove the antiaging activity of oridonin. Oridonin significantly reduced the hallmarks of cellular senescence, such as SA-β-gal positive cells in naturally senescent cells and induced senescent cells. The rate of SA-β-gal positive cells rate is considered the gold standard for cellular senescence. These results demonstrate that oridonin delays cellular senescence. At the same time, cellular senescence is considered to be one of the important causes of body aging.

Interestingly, we found that oridonin prolongs the lifespan of naturally aging mice and improves the healthy lifespan of mice, such as cognitive ability, which is also consistent with reports that oridonin can improve neurological diseases (Xu J et al., 2018). We also found that oridonin could reduce the heart and kidney damage caused by doxorubicin. The results suggest that oridonin has a protective effect on the heart and kidneys. Unfortunately, in naturally aged mice, the lifespan of mice was significantly prolonged during oridonin administration, but the lifespan extension did not last after the administration was stopped. In the premature aging mouse model, continuous oridonin administration prolonged the lifespan of mice, which suggests that oridonin needs long-term intervention to extend lifespan.

At the same time, our results indicate that oridonin works through the AKT signaling pathway. It has been reported that the AKT signaling pathway is essential for cell senescence and aging. The AKT signaling pathway can phosphorylate FOXO and transfer it from the nucleus to the cytoplasm, resulting in the downregulation of its transcriptional activity, thereby inhibiting the expression of downstream genes regulated by FOXO (Hay, 2011). The FOXO transcription factor family is involved in metabolism, apoptosis, oxidative stress resistance and aging (Hritzo Ahye and Golding, 2018). There are four FOXO proteins in mammals, namely FOXO1, FOXO3a, FOXO4, and FOXO6. FOXO1, FOXO3, and FOXO4 are widely distributed and expressed in various tissues, while FOXO6 is mainly described in nerve cells (Manning and Toker, 2017). FOXO1 and FOXO3a are nuclear proteins. As AKT is activated, FOXO1 and FOXO3a are phosphorylated, inactivated, and shuttle to the cytoplasm (Hritzo Ahye and Golding, 2018). Previous studies have shown that activation of AKT in aged zebrafish with TERT knockout leads to the translocation of FOXO1/4 into the cytoplasm, resulting in downregulation of SOD2 expression, increasing oxidative stress and mitochondrial damage, triggering p15, p16, and p21 accumulation and senescence cell cycle arrested (Seoane et al., 2004; El Mai et al., 2020). Inhibiting AKT or knocking out FOXO1 can reduce the number of senescent cells (Latorre et al., 2019). Our results confirm that oridonin inhibits AKT, thereby decreasing the phosphorylation of FOXO1 and reducing the accumulation of p21, thereby improving aging.

We found that oridonin can reduce inflammation. The aging of the immune system (called immune senescence) is one of the causes of “inflammation.” This phenomenon refers to the fact that older organisms tend to have higher levels of inflammatory factors (Franceschi et al., 2000). There are multiple triggers for systemic inflammation, but the NLRP3 inflammasome has been identified as a specific sensor that activates age-related inflammation (Youm et al., 2013). Blocking NLRP3 inflammasome assembly or activation can significantly reduce aging-related chronic inflammation (He et al., 2020). The NLRP3 inflammasome plays a vital role in the aging process of organs such as the thymus, brain, liver and kidney (Goldberg and Dixit, 2015). It can be activated by metabolic wastes, damage products, tissue remodeling events and stress factors in the aging process, regulate the activation of IL-1β and other inflammatory factors and trigger aging. IL-1β is an essential messenger between inflammation and aging (He et al., 2015). Our results also prove that oridonin reduces the activation of IL-1β inflammatory factors in senescent cells. This result indicates that oridonin also exerts anti-aging effects through NLRP3 and can potentially treat NLRP3-driven diseases, especially chronic diseases.

The RNA-seq results also show that oridonin exerts anti-aging effects through AKT and affects its downstream related signaling pathways. Studies have shown that the T cell receptor, cGMP-PKG, Hippo, and AGE-RAGE signaling pathways are also regulated by AKT (Parry et al., 2005; Kawamura et al., 2013; Zhai et al., 2014; Bao et al., 2015). There are also changes in inflammatory-related signaling pathways. These results further indicate that oridonin exerts anti-aging effects through AKT and NLRP3. It further shows that the T cell receptor, cGMP-PKG, Hippo, and AGE-RAGE signaling pathways play an essential role in aging.

## Data Availability

The datasets presented in this study can be found in online repositories. The names of the repository/repositories and accession number(s) can be found below: https://www.ncbi.nlm.nih.gov/geo/, GSE189789.
