# Supplementary material for: Oridonin Delays Aging Through the AKT Signaling Pathway
Source: Front Pharmacol. 2022 May 18;13:888247. doi: 10.3389/fphar.2022.888247 (PMC9157590; doi:10.3389/fphar.2022.888247)
Supplement: Supplementary file 1 [file DataSheet1.docx]

**Supplementary Material**

1. **Supplementary Figures**

**
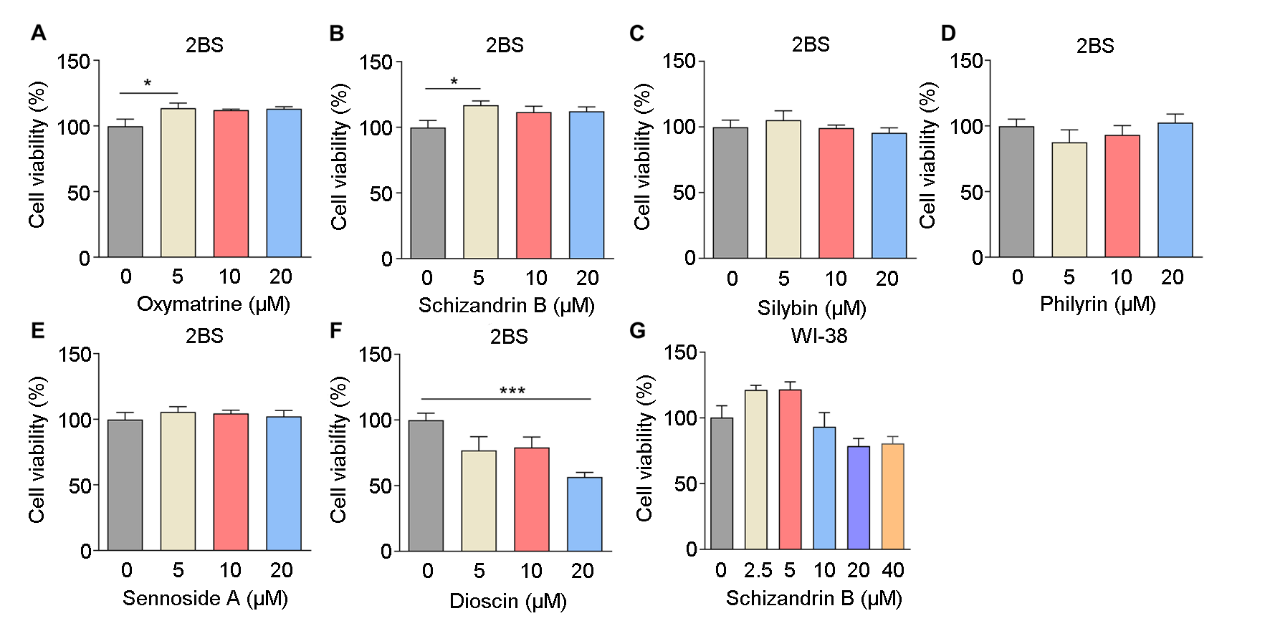
**

**Figure S1. Screening of anti-aging compounds based on CMAP**

(A) The effect of different concentrations of oxymatrine on the proliferation of PD45 2BS cells measured by the CCK-8 assay. (B) The effect of schizandrin B on the proliferation of PD45 2BS cells. (C) The effect of silybin on the proliferation of PD45 2BS cells. (D) The effect of phillyrin on the proliferation of PD45 2BS cells. (E) The effect of sennoside A on the proliferation of PD45 2BS cells. (F) The effect of dioscin on the proliferation of PD45 2BS cells. (G) The effect of schizandrin B on the proliferation of PD45 WI-38 cells (A-G, n≥ 3 for each group). Data represent the mean ± SEM. (*p<0.05, **p<0.01, ***p<0.001, ****p<0.0001).


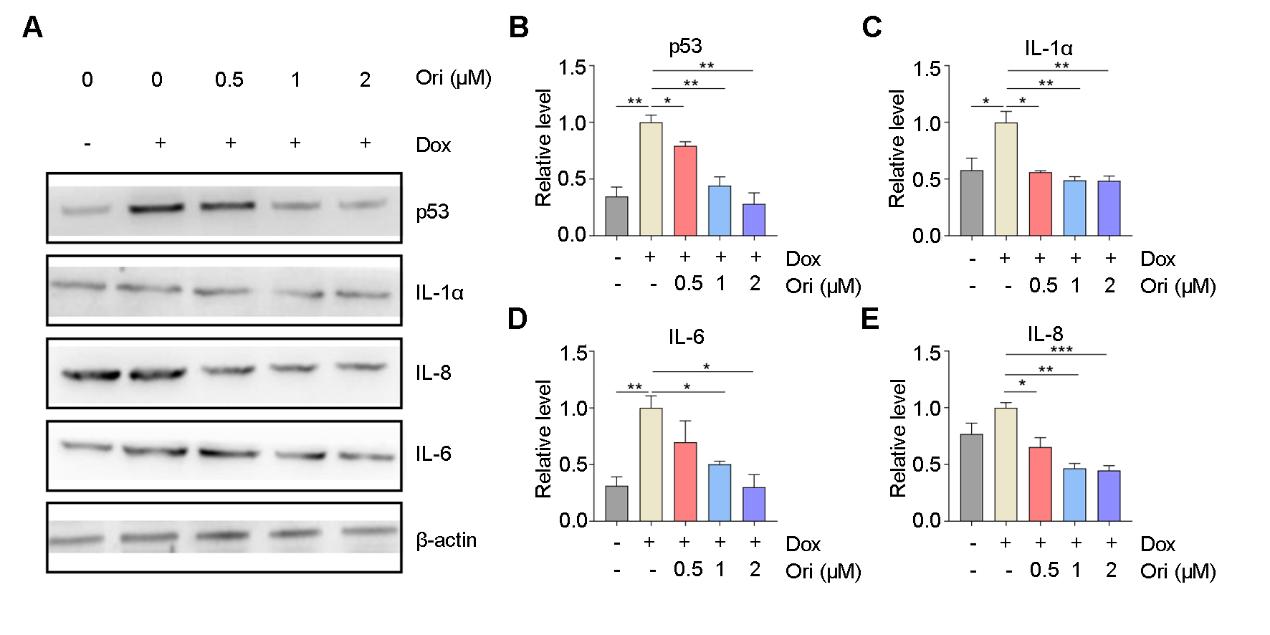


**Figure S2. Oridonin delays cell senescence induced by doxorubicin**

(A) Western blot analysis of p53, IL-1α, IL-8, and IL-6 protein expression in the control group, the doxorubicin-induced aging model group, and the 0.5, 1, 2 µM oridonin treatment group. (B-E) Quantification of p53, IL-1α, IL-8, and IL-6 protein expression in the control group, doxorubicin-induced aging model group, 0.5, 1, and 2 µM oridonin treatment group analyzed by Western blot (A-E, n=3 for each group). Data represent the mean ± SEM. p values were determined by one-way ANOVA or Student’s t test. (*p<0.05, **p<0.01, ***p<0.001, ****p<0.0001).


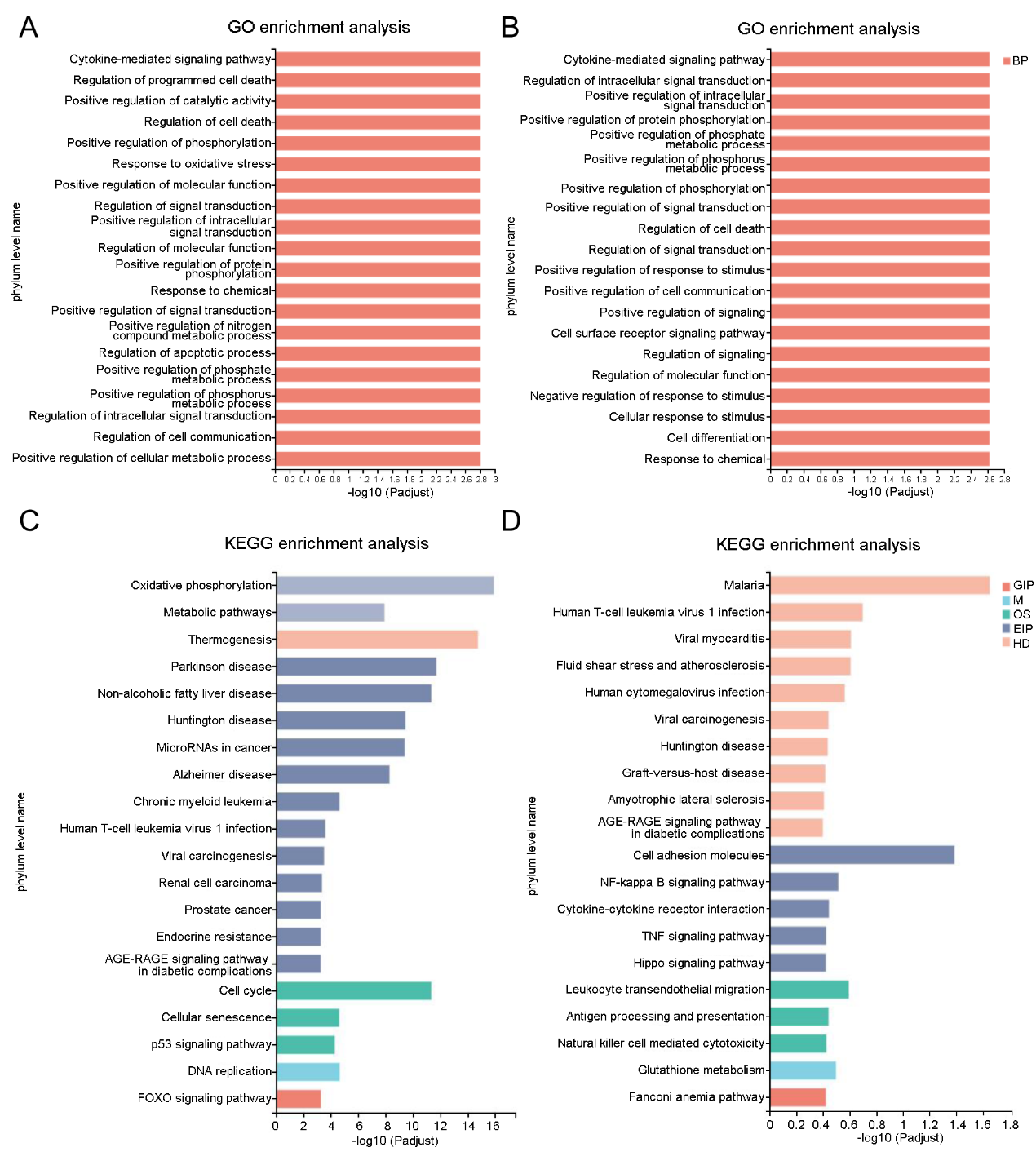


**Figure S3. Oridonin reverses the gene changes caused by doxorubicin-induced cell senescence**

WI-38 cells were first induced with 1 μM doxorubicin for 12 hours, and then treated with oridonin or DMSO-containing medium for 48 hours. (A) GO enrichment analysis: the intersection gene of the differential gene (CVSD) produced by doxorubicin-induced cell senescence and the differential gene (DVSO) produced by oridonin treatment of doxorubicin-induced senescent cells. Biological process (BP). (B) GO enrichment analysis: the differential gene (DVSO) produced by oridonin treatment of doxorubicin-induced senescent cells. Biological process (BP). (C) KEGG enrichment analysis: the differential gene (CVSD) produced by doxorubicin-induced cell senescence. (D) KEGG enrichment analysis: the differential gene (DVSO) produced by oridonin treatment of doxorubicin-induced senescent cells. Different colors indicate the 7 branches of the KEGG metabolic pathway, namely metabolism (M), genetic information processing (GIP), environmental information processing (EIP), cellular processes (CP), biological systems (OS), and human diseases (HD), Drug Development (DD). (A-D, n = 3 for each group). Data represent the mean ± SEM. p values were determined by one-way ANOVA or Student’s t test. (*p<0.05, **p<0.01, ***p<0.001, ****p<0.0001).

1. **Supplementary Tables**

| Primer sequences used in qPCR | |
| --- | --- |
| Homo sapiens | |
| Gene | Primer sequence（5’ to 3‘） |
| GAPDH FOR | CAGCAAGTGGGAAGGTGTAATCC |
| GAPDH REV | CCCATTCTATCATCAACGGGTACAA |
| IL-1α FOR | AGTAGCAACCAACGGGAAGG |
| IL-1α REV | TGGTTGGTCTTCATCTTGGG |
| IL-1β FOR | ACAGATGAAGTGCTCCTTCCA |
| IL-1β REV | GTCGGAGATTCGTAGCTGGAT |
| IL-6 FOR | GGTACATCCTCGACGGCATCT |
| IL-6 REV | GTGCCTCTTTGCTGCTTTCAC |
| IL-8 FOR | ATGACTTCCAAGCTGGCCGTGGCT |
| IL-8 REV | TCTCAGCCCTCTTCAAAAACTTCT |
| P53 FOR | TCAGCATCTTATCCGAGTGGAA |
| P53 REV | TGTAGTGGATGGTGGTACAGTCA |
| p21 FOR | TGTCCGTCAGAACCCATGC |
| P21 REV | AAAGTCGAAGTTCCATCGCTC |
| PTPRC FOR | ATTACCTGGAATCCCCCTCAAA |
| PTPRC REV | TTGTGAAATGACACATTGCAGC |
| LAMTOR4 FOR | TCTTGGCTTGCTGCTAGAACT |
| LAMTOR4 REV | CACAGCTCAGAGTCCCCAAG |
| H4C14 FOR | GAAAAGGCTTAGGCAAAGGGG |
| H4C14 REV | CCAGAGATCCGCTTAACGC |
| ZFP91 FOR | TGAGACCTACAAACCCCACTT |
| ZFP91 REV | CCTTTTGGGTAAACGTGGACTTT |
